# Proper timing or ERCP and cholecystectomy on acute cholecystitis: a systematic review and meta-analysis

**DOI:** 10.1590/acb401025

**Published:** 2025-01-13

**Authors:** Giuliana Fulco Gonçalvez, Louise Lopes Barros, Sofia Emereciano Gurgel, Kleyton Santos de Medeiros, Irami Araújo

**Affiliations:** 1 Instituto de Ensino, Pesquisa e Inovação – Liga Contra o Câncer – Natal (RN) – Brazil.; 2 Universidade Potiguar – Department of Medicine – Natal (RN) – Brazil.; 3 Universidade Federal do Rio Grande do Norte – Postgraduate Program in Health Sciences – Natal (RN) – Brazil.; 4 Universidade Federal do Rio Grande do Norte – Department of Surgery – Natal (RN) – Brazil.

**Keywords:** Cholangiopancreatography, Endoscopic Retrograde, Cholecystectomy, Cholecystitis, Acute

## Abstract

**Purpose:**

To determine if endoscopic retrograde cholangiopancreatography (ERCP) should be performed with surgery or as a different step, on acute cholecystitis, and which strategy has the least complications and morbimortality.

**Methods:**

Various databases (PubMed, Embase, Scopus, Web of Science, Science Direct, Cochrane Central Register of Controlled Trials, CINAHL, Latin American and Caribbean Health Sciences Literature, clinical trials, Google Scholar) were searched for randomized trials comparing the different timings for ERCP and cholecystectomy. No language or time restrictions were applied. Risk of bias was assessed with RoB 2.0 (Cochrane’s Risk of Bias 2), and evidence certainty evaluated using Grading of Recommendations Assessment, Development and Evaluation. Data synthesis used R-4.1.0 Project for Statistical Computing for Windows, with meta-analysis via fixed-effects model and I2 for heterogeneity.

**Results:**

Eleven studies was used, and meta-analysis was performed independently for each outcome. Different outcomes were evaluated, with preoperative ERCP as an intervention and intraoperative ERCP as the control: length of stays (four trials with mean differences – MD = -1.44; 95% confidence interval – 95%CI -3,87–0,98); bile leak (*odds ratio* – OR = 0.67; 95%CI 0.11–4.09); cholangitis (OR = 1.32; 95%CI 0.29–5.98); bleeding from sphincterotomy (OR = 0.98; 95%CI 0.20–4.86); wound infection (OR = 0.33; 95%CI 0.04–3.14); incisional bleeding (OR = 0.5; 95%CI 0.04–5.70); elevated amylase activity (OR = 5.22; 95%CI 2.17–12.59); acute pancreatitis (OR = 4.61; 95%CI 1.72–12.38); operative time (MD = -6,26; 95%CI -37.24–24.73); failure rate (OR = 1.74; 95%CI 0.99–3.05); conversion (OR = 1.34; 95%CI 0.6–2.96); morbidity (OR = 2.75; 95%CI 1.7–4.47).

**Conclusions:**

Risk of bias was significant due to lack of blindness. The morbidity, pancreatitis, and elevated amylase activity outcomes were the only ones to find statistical significance and favored the intraoperative approach.

## Introduction

Cholangiopancreatography retrograde endoscopy (ERCP) is a medical procedure performed with the purpose of diagnosing and treating diseases of the biliary tract, similar to cholecystectomy. ERCP is an endoscopic intervention that allows access to the biliary and pancreatic ducts. However, although it has a dual purpose, it is more often used for treatment than for diagnosis, as there are highly accurate diagnostic methods that do not expose the patient to the risks associated with ERCP. Among the potential adverse events associated with this procedure, post-ERCP pancreatitis is the most common complication, and the failure rate in cannulating the Vater’s ampulla ranges from 4 to 18%^1^^,^ 2.

Furthermore, cholecystectomy is the standard surgery for treating gallbladder diseases^3^. This procedure involves the complete removal of the gallbladder and is primarily performed laparoscopically. Laparoscopic surgery, on the other hand, is associated with fewer cases of surgical trauma^4^.

In this perspective, the surgical outcomes related to the combination of both surgeries in a complementary manner are not yet well understood, despite this association already being put into practice^5^. Thus, considering that the techniques in question have similar reasons for being performed, we sought to analyze the impact of early ERCP execution in patients with acute cholecystitis subsequently undergoing cholecystectomy procedures, evaluating whether there is an improvement in morbidity rates, comparing the different timing options (before, simultaneously, or after) between the execution of ERCP and cholecystectomy.

## Methods

### Study design

The review was carried out in accordance with the Preferred Reporting Items for Systematic Reviews and Meta-Analysis (PRISMA)6 and the Cochrane Collaboration’s^7^ reporting standards for systematic reviews and meta-analysis. A review protocol was developed and registered on the Prospective Register of Systematic Reviews (PROSPERO), with the registration reference CRD42021290726. Additionally, the protocol was previously published in a scientific journal^8^.

### Review questions

- Is it better to perform ERCP intraoperatively or before the cholecystectomy, in terms of general complications?;
- Which approach is more likely to convert into a cholecystectomy?;
- Which approach is more cost effective?

The questions were formulated based on the PICOS framework, and the elements were as it follows:

- Population: patients with acute cholecystitis;
- Intervention: ERCP before surgery;
- Comparison: intraoperative ERCP;
- Outcome: morbidity, with length of stays, conversions, operative time, failure rate, acute pancreatitis, cholangitis, wound infection, incisional bleeding, sphincterotomy bleeding, bile leakage, and elevated amylase activity;

- Study design: randomized clinical trials.

### Eligibility criteria

The systematic review encompassed randomized clinical trials that examined outcomes related to cholecystectomy and the timing of ERCP. The inclusion criteria stipulated that studies must involve adult patients aged 18 years old or older diagnosed with acute cholecystitis. The review did not impose any restrictions based on language or publication date. Case series, case reports, cohorts, and pre-clinical trials were excluded from this review, as well as studies that did not meet the specified inclusion criteria.

### Outcome measures

This review evaluated multiple outcomes, such as:

- Length of stay (hospitalization);
- Bile leakage;
- Cholangitis;
- Bleeding from sphincterotomy;
- Incisional bleeding;
- Wound infection;
- Elevated amylase activity;
- Acute pancreatitis;
- Operative time;
- Failure rate;
- Conversions;
- Morbidity.

### Data searches and sources

A comprehensive search was performed across various databases, employing Medical Subject Headings (MeSH) search terms, text words, and database-specific keywords. The search focused on identifying terms related to ERCP and cholecystectomies. The databases included in this search were PubMed, Embase, Scopus, Web of Science, Science Direct, Latin American and Caribbean Health Sciences Literature, Cochrane Central Register of Clinical Trials, CINAHL, as well as clinical trial databases (www.trialscenter.org, www.controlled-trials.com, and www.clinicaltrials.gov).

Our keyword search was based on MeSH conform to the combination: (Choledocholithiasis OR “Common Bile Duct” OR “biliary obstruction” OR Gallstones OR Cholelithiasis OR “Cholecystitis, acute” OR Cholecystitis) AND (Cholangiopancreatography OR “Endoscopic Retrograde” OR “ERCP” OR Endoscopy OR Cholangiography OR “Sphincterotomy, Endoscopic” OR “Endoscopic Papillotomy” OR “Endoscopic Papillotomies” OR “Biliary Tract Surgical Procedures”) AND (Cholecystectomy OR “Cholecystectomy, Laparoscopic” OR “Laparoscopic Cholecystectomy”) AND (“Postoperative Complications” OR “Pain, Postoperative” OR “Post-surgical Pain” OR “Post Cholecystectomy Syndrome” OR Hospitalization OR Infections OR “Stay length” OR Fever OR “Incisional Hernia”) AND (randomized controlled trials OR Controlled Clinical Trial). The searches were consolidated using the review tool Rayyan.

### Data extraction

For every eligible study, two independent reviewers performed data extraction utilizing a standardized data extraction form. In cases of discrepancies between the two reviewers, a third reviewer was consulted to reach a consensus through discussion. The extracted information from each included study encompassed the following elements: author(s), publication year, study design, sample size, details of the intervention, outcome measures, and pertinent findings. The primary outcome of interest was morbidity, with length of stays, conversions, operative time, failure rate, acute pancreatitis, cholangitis, wound infection, incisional bleeding, sphincterotomy bleeding, bile leakage, and elevated amylase activity being considered as secondary outcomes.

### Risk of bias assessment

Two evaluators conducted separate assessments of study quality, employing the Cochrane Collaboration’s Risk of Bias Tool for Randomized Controlled Trials^9^. Each study underwent a comprehensive evaluation for possible biases across essential domains, including the random sequence generation, allocation concealment, blinding of both participants and personnel, blinding of outcome assessment, handling of incomplete outcome data, and potential selective reporting. Any discrepancies in their assessments were addressed through a consensus discussion involving a third reviewer.

### Grade certainty of evidence

The quality of evidence underwent assessment using the Grading of Recommendations, Assessment, Development, and Evaluation (GRADE)^10^ approach, with a specific focus on primary outcomes and the occurrence of serious adverse events.

### Data synthesis

A meta-analysis utilized the R Project for Statistical Computing software (version R-4.3.1)^11^. Each randomized clinical trial included in the analysis reported continuous outcomes as either mean ± standard deviation, mean differences (MD), standardized mean differences (SMD), or hazard ratios (HR), while dichotomous outcomes were expressed as *odds ratios* (OR) using Mantel-Haenszel random-effects analysis, accompanied by 95% confidence intervals (95%CI) for all outcome measurements. Heterogeneity among the studies was quantified using Cochrane’s Q test, and inconsistency was assessed with the I2 test. When the I2 value fell in the range of 0–50%, acceptable heterogeneity was considered to be present.

## Results

### Recruitment process

**Figure 1:**
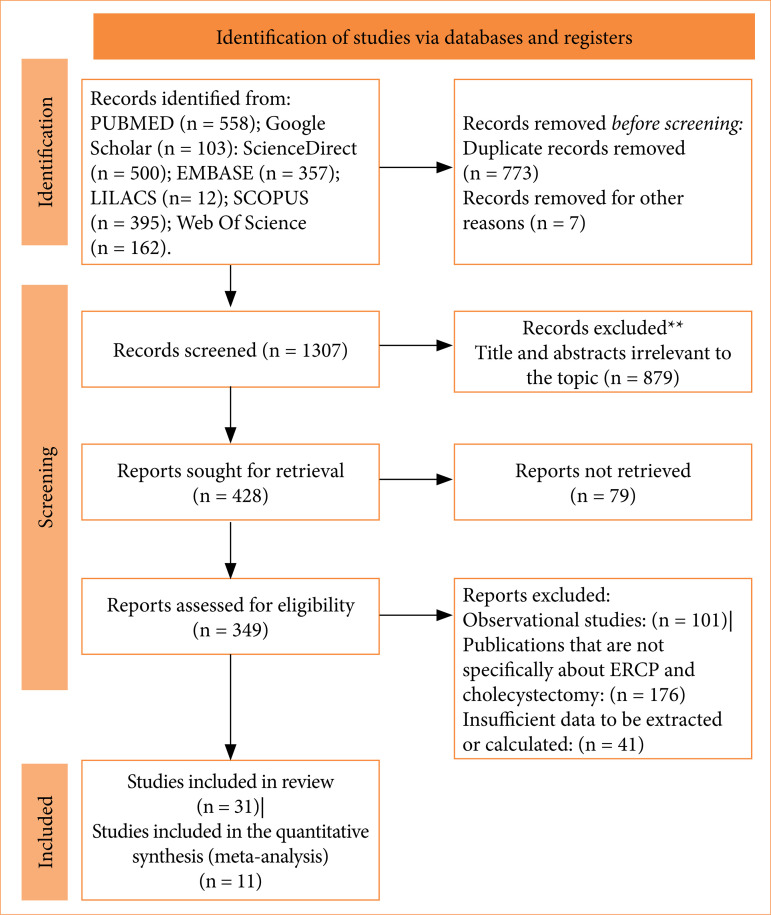
Preferred Reporting Items for Systematic Reviews and Meta-Analysis (PRISMA) flow diagram for systematic review and meta-analysis

Source: Elaborated by the authors.

The initial electronic search yielded a total of 2,087 studies before the screening process. Following the removal of duplicates, 773 records were eliminated, and an additional seven were excluded. Among the remaining 1,307 articles, 879 titles and/or abstracts were deemed irrelevant to the study. Then, 428 reports were sought for retrieval, with 79 not being retrieved. Then, out of the 349 studies assessed for eligibility, 101 were excluded for being observational studies, 176 for not having ERCP and cholecystectomy as their main topic, and 41 were excluded for their data being insufficient to be extracted or calculated. After reviewing titles, abstracts, and full-texts, 31 studies met the inclusion criteria and were selected to compose the review, and 11 were used to generate the meta-analyses (Fig. 1).

### Study characteristics

The meta-analysis incorporated randomized controlled trials released from 2000 to 2017, focusing on patients with acute cholecystitis who underwent a comparison between preoperative ERCP and intraoperative ERCP (as depicted in Table 1). The included studies had sample sizes ranging from 20 to 198 participants, amounting to a combined total of 1,069 participants across all of them.

**Table 1:** Selected randomized controlled trials.

Reference	Year	Type	N. of patients	Country	Sex (F/M)	Mean Age (two-step)	Mean Age (one-step)
Tzovaras et al.^2^	2012	RCT	99	Greece	50F/49M	69 (25–58)	66 (22–87)
Jones et al.^3^	2013	RCT	20	USA	19F/01M	41.1	38.6
Liu et al.^5^	2017	RCT	63	China	21F/42M	40 (+6.1)	42 (+5.2)
Morino et al.^12^	2006	RCT	91	Italy	56F/35M	63.1 (25–85)	56.6 (22–82)
Lella et al.^13^	2006	RCT	120	Italy	68F/52M	54.2 (22–60)	(Both groups)
Rábago et al.^14^	2006	RCT	123	Spain	-	-	-
González et al.^15^	2015	RCT	134	Cuba	-	57.7 (20–84)	58.4 (23–87)
ElGeidie et al.^16^	2011	RCT	198	Egypt	144F/54M	27.5	31.2
Chang et al.^17^	2000	RCT	59	USA	-	39 (+14.7)	38.4 (+13.8)
Salman et al.^18^	2009	RCT	79	Turkey	54F/36M	43.5 (+8.6)	44.6 (+3.2)
Sahoo et al.^19^	2014	RCT	83	India	53F/30M	47.95	(Both groups)

RCT: randomized clinical trial. Source: Elaborated by the authors.

### Qualitative analysis

Amidst the trials, only one provided information regarding pulmonary emboli, drain site hemorrhage, collections, and urinary retention. The same was noted for fever and pulmonary infection. Sepsis was not an outcome evaluated on these trials. Patients age, sex, and ASA score was an information provided by some trials, although not discussed in this review. Pancreatitis and bile leak were evaluated by two trials.

### Length of stay

**Figure 2:**
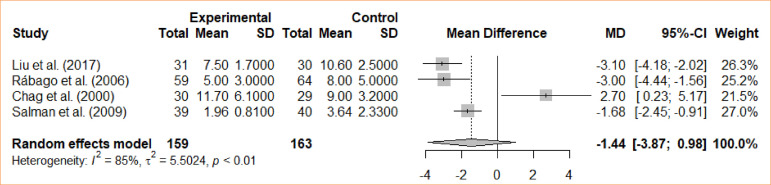
Forest plot for length of stay

SD: standard deviation; MD: mean difference; 95%CI: 95% confidence interval. Source: Elaborated by the authors.

A random-effects model was used for the analysis. Four studies were considered for the comparative analysis. The analyzed studies did not show statistical differences between intraoperative cholecystectomy and preoperative cholecystectomy for the outcome in question (MD = -1.44; 95%CI -3,87–0.98). The meta-analysis did not find a statistically significant difference for the groups either (Fig. 2).

### Bile leak

**Figure 3:**
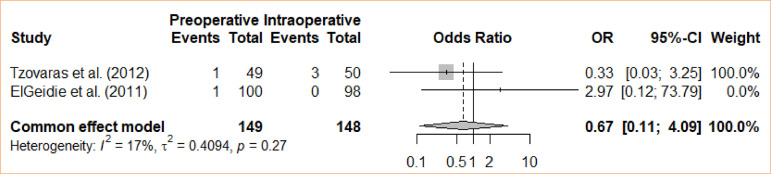
Forest plot for bile leak.

The analysis involved a comparison between intraoperative cholecystectomy and preoperative cholecystectomy in terms of bile leakage. The meta-analysis, using a fixed-effects model, did not reveal a statistically significant difference for the groups concerning bile leakage (OR = 0.67; 95%CI 0.11–4.09) (Fig. 3).

OR: *odds ratio*; 95%CI: 95% confidence interval. Source: Elaborated by the authors.

### Cholangitis

**Figure 4:**
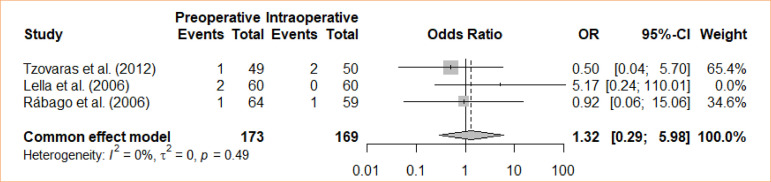
Forest plot for cholangitis.

The analysis examined the comparison between intraoperative cholecystectomy and preoperative cholecystectomy in relation to cholangitis. The meta-analysis, using a fixed-effects model, did not reveal a statistically significant difference between the groups concerning cholangitis (OR = 1.32; 95%CI 0.29–5.98) (Fig. 4).

OR: *odds ratio*; 95%CI: 95% confidence interval. Source: Elaborated by the authors

### Sphincterotomy bleeding

**Figure 5:**
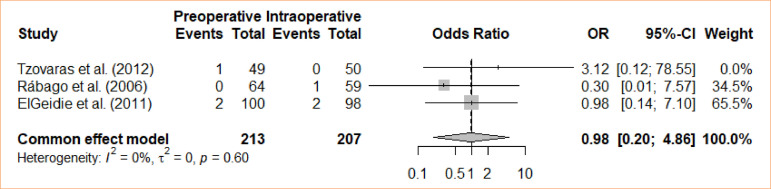
Forest plot for sphincterotomy bleeding.

In the study, the comparison between intraoperative cholecystectomy and preoperative cholecystectomy with respect to sphincterotomy-induced bleeding was investigated. The meta-analysis, employing a fixed-effects model, did not identify any statistically significant differences in the groups regarding bleeding caused by sphincterotomy (OR = 0.98; 95%CI 0.20–4.86) (Fig. 5).

OR: *odds ratio*; 95%CI: 95% confidence interval. Source: Elaborated by the authors.

### Wound infection

**Figure 6:**
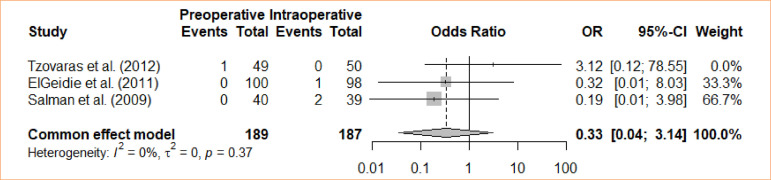
Forest plot for wound infection.

A comparative analysis was conducted between intraoperative cholecystectomy and preoperative cholecystectomy regarding wound infection. Through a meta-analysis and using the fixed-effects model, no statistically significant difference was found between the groups concerning wound infection (OR = 0.33; 95%CI 0.04–3.14) (Fig. 6).

OR: *odds ratio*; 95%CI: 95% confidence interval. Source: Elaborated by the authors.

### Incisional bleeding

**Figure 7:**
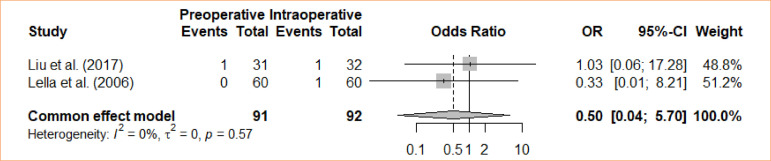
Forest plot for incisional bleeding.

An analysis was conducted to compare intraoperative cholecystectomy and preoperative cholecystectomy in terms of incisional bleeding. Through a meta-analysis and the utilization of the fixed-effects model, no statistically significant distinction was discovered between the groups in relation to incisional bleeding (OR = 0.5; 95%CI 0.04 to 5.70) (Fig. 7).

OR: *odds ratio*; 95%CI: 95% confidence interval. Source: Elaborated by the authors.

### Elevated amylase activity

**Figure 8:**
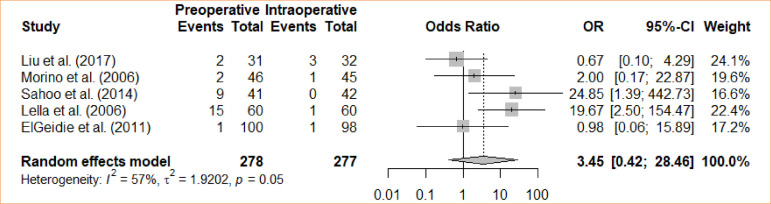
Forest plot for elevated amylase activity.

A comparative assessment was undertaken to examine the difference in postoperative elevated amylase activity between intraoperative cholecystectomy and preoperative cholecystectomy. Through a meta-analysis, while employing the fixed-effects model, a statistically significant variance was observed among the groups concerning the heightened amylase activity following the procedure, favoring the intraoperative approach (OR = 3.45; 95%CI 0.42–28.46) (Fig. 8).

OR: *odds ratio*; 95%CI: 95% confidence interval. Source: Elaborated by the authors.

### Acute pancreatitis

**Figure 9:**
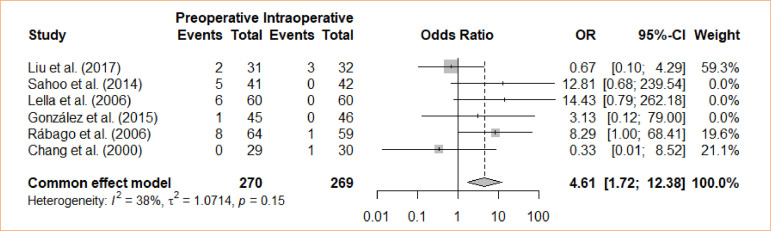
Forest plot for acute pancreatitis.

A comparison was made between intraoperative cholecystectomy and preoperative cholecystectomy in relation to acute pancreatitis. Through a meta-analysis, using the fixed-effects model, a statistically significant difference was identified between the groups regarding the occurrence of acute pancreatitis, favoring the intraoperative approach (OR = 4.61; 95%CI 1.72–12.38) (Fig. 9).

OR: *odds ratio*; 95%CI: 95% confidence interval. Source: Elaborated by the authors.

### Operative time

**Figure 10:**
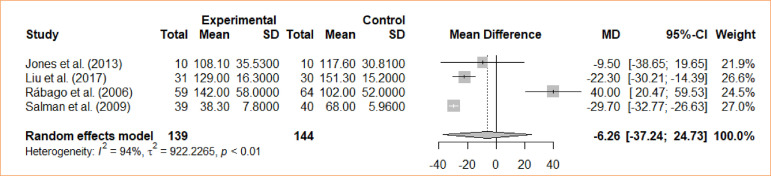
Forest plot for operative time.

SD: standard deviation; MD: mean difference; 95%CI: 95% confidence interval. Source: Elaborated by the authors.

An evaluation was conducted to compare intraoperative cholecystectomy and preoperative cholecystectomy in relation to operative time. A meta-analysis, utilizing the random-effects model, revealed no statistically significant difference between the groups regarding the duration of the operation (MD = -6,26; 95%CI -37.24–24.73) (Fig. 10).

### Failure rate

**Figure 11:**
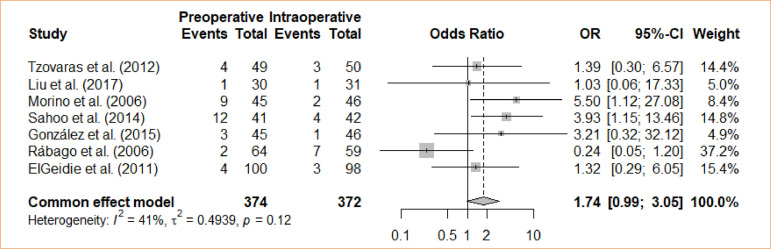
Forest plot for failure rate.

An assessment was made regarding the comparison of intraoperative cholecystectomy and preoperative cholecystectomy concerning the failure rate. Through a meta-analysis, using the fixed-effects model, no statistically significant distinction was identified between the groups in relation to the failure rate (OR = 1.74; 95%CI 0.99–3.05) (Fig. 11).

OR: *odds ratio*; 95%CI: 95% confidence interval. Source: Elaborated by the authors.

### Conversion

**Figure 12:**
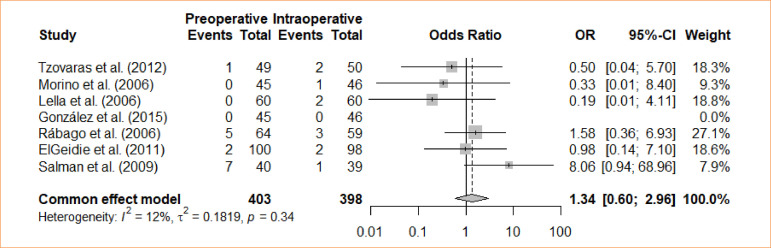
Forest plot for conversion.

An analysis was conducted to compare intraoperative cholecystectomy and preoperative cholecystectomy regarding the conversion rate. Through a meta-analysis, using the fixed-effects model, no statistically significant distinction was identified between the groups with respect to conversion (OR = 1.34; 95%CI 0.6–2.96) (Fig. 12).

OR: *odds ratio*; 95%CI: 95% confidence interval. Source: Elaborated by the authors.

### Morbidity

**Figure 13:**
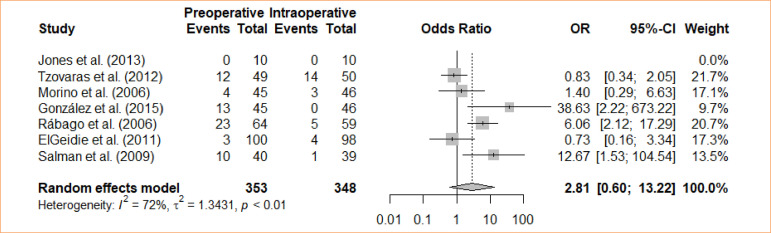
Forest plot for morbidity.

The studies evaluated the comparison between intraoperative cholecystectomy and preoperative cholecystectomy in relation to morbidity. A meta-analysis, using the fixed-effects model, revealed a statistically significant difference between the groups concerning morbidity (OR = 2.81; 95%CI 0.60–13.22) (Fig. 13).

OR: *odds ratio*; 95%CI: 95% confidence interval. Source: Elaborated by the authors.

### Risk of bias

**Table 2:** Risk of bias for each trial*.

Study/Year Reference	Random sequence generation	Allocation concealment	Blinding of participants and personnel	Blinding of outcome assessment	Incomplete outcome data	Selective reporting	Other bias
Barreras González et al. (2015)	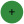	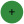	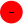	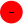	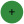	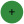	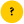
Rabago et al. (2006)	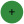	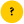	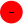	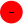	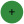	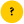	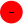
ElGeidie et al. (2011)	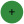	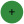	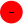	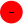	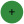	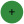	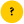
Chang et al. (2000)	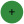	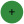	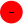	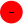	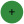	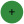	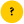
Salman et al. (2009)	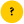	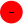	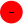	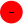	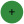	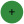	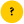
Jones et al. (2013)	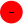	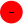	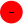	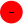	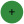	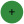	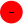
Tzovaras et al. (2012)	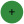	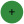	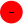	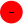	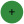	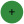	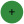
Liu et al. (2017)	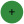	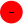	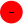	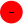	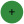	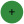	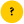
Morino et al. (2006)	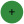	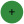	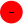	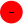	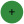	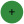	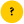
Sahoo et al. (2014)	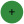	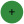	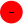	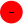	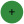	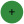	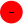
Lella et al. (2006)	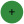	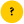	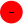	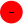	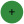	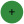	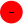

* Two displays the risk of bias for each trial.

Overall, all the studies exhibited some level of reporting bias. This is attributed to the nature of the procedures, as it is not feasible to achieve allocation concealment and blinding of all participants and personnel. Source: Elaborated by the authors.

### Certainty of evidence

Table 3 provides an overview of the quality and confidence level of evidence for every outcome assessed in the clinical trials. The primary outcome, which is postoperative infection, and the other outcome analyzed in the meta-analysis have been categorized as having a low certainty of evidence in accordance with the GRADE guidelines.

**Table 3:** Quality and confidence level of evidence for every outcome assessed in the clinical trials.

Certainty assessment								N. of patients			Effect		Certainty	Importance
Studies	Study design	Risk of bias	Inconsistency	Indirectness	Imprecision	Other considerations		ERCP preoperative	ERCP simultaneously		Relative (95%CI)	Absolute (95%CI)		
**Hospital length of stay**														
4	randomised trials	serious	not serious	not serious	not serious	none		159	163		-	MD **1.44 days lower** (3.87 lower to 0.98 higher)	⨁⨁⨁◯ Moderate	IMPORTANT
**Bile leakage**														
2	randomised trials	seriousa	not serious	not serious	not serious	none		2/149 (1.3%)	3/148 (2.0%)		**OR 0.67** (0.11 to 4.09)	**7 fewer per 1,000** (from 18 fewer to 58 more)	⨁⨁⨁◯ Moderate	IMPORTANT
**Cholangitis**														
3	randomised trials	serious	not serious	not serious	not serious	none		4/173 (2.3%)	3/169 (1.8%)		**OR 1.32** (0.29 to 5.98)	**6 more per 1,000** (from 13 fewer to 80 more)	⨁⨁⨁◯ Moderate	IMPORTANT
**Sphincterotomy bleeding**														
3	randomised trials	serious	not serious	not serious	not serious	none		3/213 (1.4٪)	3/207 (1.4٪)		**OR 0.98** (0.20 to 4.86)	**0 fewer per 1,000** (from 12 fewer to 52 more)	⨁⨁⨁◯ Moderate	IMPORTANT
**Wound infection**														
3	randomised trials	serious	not serious	not serious	not serious	none		1/189 (0.5٪)	3/187 (1.6٪)		**OR 0.33** (0.04 to 3.14)	**11 fewer per 1,000** (from 15 fewer to 33 more)	⨁⨁⨁◯ Moderate	IMPORTANT
**Postoperative bleeding**														
2	randomised trials	serious	not serious	not serious	not serious	none		1/91 (1.1٪)	2/92 (2.2٪)		**OR 0.50** (0.04 to 5.70)	**11 fewer per 1,000** (from 21 fewer to 91 more)	⨁⨁⨁◯ Moderate	IMPORTANT
**Elevated amilase activity**														
5	randomised trials	serious	not serious	not serious	not serious	none		29/278 (10.4٪)	6/277 (2.2٪)		**OR 5.22** (2.17 to 12.59)	**82 more per 1,000** (from 24 more to 196 more)	⨁⨁⨁◯ Moderate	IMPORTANT
**Acute pancreatitis**														
6	randomised trials	serious	not serious	not serious	not serious	none		22/270 (8.1٪)	5/269 (1.9٪)		**OR 4.61** (1.72 to 12.38)	**62 more per 1,000** (from 13 more to 171 more)	⨁⨁⨁◯ Moderate	IMPORTANT
**Operative time**														
4	randomised trials	serious	not serious	not serious	not serious	none		139	144		-	MD **6.26 hours lower** (6.26 lower to 24.73 higher)	⨁⨁⨁◯ Moderate	IMPORTANT
**Failure rate**														
7	randomised trials	serious	not serious	not serious	not serious	none		35/374 (9.4٪)	21/372 (5.6٪)		OR 1.74 (0.99 to 3.05)	38 more per 1,000 (from 1 fewer to 98 more)	⨁⨁⨁◯ Moderate	IMPORTANT
**Conversion**														
7	randomised trials	serious	not serious	not serious	not serious	none		15/403 (3.7٪)	11/398 (2.8٪)		**OR 1.34** (0.60 to 2.96)	**9 more per 1,000** (from 11 fewer to 50 more)	⨁⨁⨁◯ Moderate	IMPORTANT
**Morbidity**														
7	randomised trials	serious	not serious	not serious	not serious	none		65/353 (18.4٪)	27/348 (7.8٪)		OR 2.75 (1.70 to 4.47)	110 more per 1,000 (from 48 more to 196 more)	⨁⨁⨁◯ Moderate	IMPORTANT

ERCP: endoscopic retrograde cholangiopancreatography; 95%CI: 95% confidence interval; MD: mean difference; OR: *odds ratio*; anature of surgery does not allow complete blindness. Source: Elaborated by the authors.

## Discussion

In this systematic review, we examined the comparative effectiveness of both surgical interventions in various clinical aspects. The review encompassed a comprehensive analysis of multiple clinical trials and their respective outcomes, shedding light on key aspects of these surgical approaches.

Our analysis revealed that, in the comparison between intraoperative cholecystectomy and preoperative cholecystectomy, several clinical parameters were assessed. These parameters included wound infection, incisional bleeding, elevated amylase activity, acute pancreatitis, operative time, conversion rate, morbidity, and the certainty of evidence for each outcome. The systematic review employed meta-analyses and different statistical models to evaluate the significance of the findings.

It was observed that, concerning wound infection and incisional bleeding, no statistically significant differences were found between intraoperative ERCP and pre-cholecystectomy ERCP. This suggests that both approaches have similar outcomes in these aspects.

For acute pancreatitis and amylase activity, our analysis indicated a statistically significant difference between the two surgical approaches, with a higher occurrence of acute pancreatitis and amylase activity associated with the preoperative approach, favoring the intraoperative one.

Operative time, an important factor in surgical decision-making, was found to be statistically equivalent between intraoperative ERCP and preoperative ERCP. This information is vital for clinicians and patients when considering the duration of surgery.

Conversion rate, a critical parameter, revealed no statistically significant difference between the two approaches, providing evidence that both methods can be considered with a similar risk of conversion.

The assessment of morbidity showed a statistically significant difference between the groups, suggesting that one of the approaches may be associated with a higher morbidity rate. Further details regarding which approach exhibited a higher morbidity rate would be included based on the actual data presented in the results section.

The certainty of evidence for each outcome, as per GRADE guidelines, was moderate for the primary outcome of postoperative infection and the other meta-analysis outcome. This indicates that the quality of evidence supporting these outcomes may have limitations that affect the confidence in the findings.

It is important to note that, while this systematic review provides valuable insights into the comparative effectiveness of preoperative ERCP and intraoperative ERCP, the specific choice of procedure should be made in consultation with a healthcare professional, taking into consideration the individual patient’s medical history and circumstances.

Initially, Jones et al.^3^ analyzed that, despite the length of hospitalization and preoperative days not having significant relevance when comparing the single-stage procedure with the two-stage procedure, the cost of the former is significantly more economical than the latter.

From another perspective, Tzovaras et al.^2^ concluded that the single-stage approach, utilizing the laparoscopic rendezvous technique, is superior as it is associated with a shorter hospitalization time compared to the conventional two-stage technique. Furthermore, despite not showing clinical significance, the first technique had a greater association with lower amylase levels post-procedure.

Liu et al.^5^, on the other hand, considers the single-stage procedure as more advantageous, with the two-stage involving ERCP and endoscopic sphincterotomy being related to shorter hospitalization time and lower association with pulmonary infection.

In contrast, Morino et al.^12^, in their study, compared the use of CPRE associated with endoscopic sphincterotomy with the technique of CPRE followed by endoscopic sphincterotomy associated with laparoscopic cholecystectomy. Consequently, they found that the second technique is superior, showing a higher rate of stone elimination, shorter hospitalization time, and reduced costs.

In Lella et al.^13^ study, two groups were separated, with the first treated in a single stage with videolaparoscopic cholecystectomy, intraoperative cholangiography, and endoscopic sphincterotomy during the surgical procedure. The second group underwent preoperative CPRE and endoscopic sphincterotomy. They concluded that there was low morbidity, a short period of hospitalization, and favorable outcomes in gallbladder obstruction, particularly in the first group.

In their study, Muhammedoğlu and Kale^4^ compared three groups, with the first undergoing CPRE and laparoscopic cholecystectomy in the same session, the second using the same method during the same hospitalization period, and a third group (delayed) consisting of patients undergoing CPRE followed by laparoscopic cholecystectomy in six to eight weeks. From this, it can be observed that the first two groups had an advantage due to the absence of the risk of recurrent episodes of acute cholecystitis associated with delayed cholecystectomy.

Moreover, Rábago et al.^14^ compared intraoperative CPRE during laparoscopic cholecystectomy with preoperative CPRE followed by laparoscopic cholecystectomy, concluding that the intraoperative CPRE group had less morbidity, shorter hospitalization time, reduced costs and lower morbidity^15^.

Additionally, ElGeidie et al.^16^ separated the groups into preoperative endoscopic sphincterotomy followed by laparoscopic cholecystectomy and laparoscopic cholecystectomy combined with intraoperative endoscopic sphincterotomy. From this, they affirmed that performing laparoscopic cholecystectomy and intraoperative CPRE reduces hospitalization and costs.

On the other hand, Chang et al.^17^ analyzed routine preoperative CPRE followed by laparoscopic cholecystectomy compared to laparoscopic cholecystectomy with selective postoperative CPRE and endoscopic sphincterotomy only if a common bile duct stone was present in the common bile duct. The included patients, in conclusion of the study, can safely undergo laparoscopic cholecystectomy without preliminary CPRE.

Furthermore, Salman et al.^18^ compared different times of performing CPRE before the operation. The first group had CPRE done 24 to 72 hours before, while the second group had it more than 72 hours before the operation, with the aim of evaluating the effects of the time interval between CPRE and laparoscopic cholecystectomy. Despite the small sample size, this study suggested that performing LC in the first 72 hours yields better results, as inflammation in longer periods makes the operation more challenging.

Thus, it is valid to emphasize that the experience of the medical team and the resources of the procedural service are of great importance in choosing the most suitable technique for each situation.

## Conclusion

This systematic review underscores the importance of a thorough evaluation of clinical evidence to guide decision-making in surgical procedures. The findings here can serve as a valuable resource for clinicians and researchers, but it is essential to consider the specific clinical context and patient factors when making decisions about cholecystectomy approaches. The benefit of choosing the intraoperative approach seems limited to amylase elevation and acute pancreatitis, with the results for other outcomes not reaching statistical significance. Future research should continue to refine our understanding of these surgical interventions to enhance patient outcomes.

## Data Availability

All data sets were generated or analyzed in the current study.
